# Management of Recurrent Temporomandibular Joint Dislocation in Children: A Systematic Review

**DOI:** 10.3390/jcm14217881

**Published:** 2025-11-06

**Authors:** Amelia Hoppe, Natalia Turosz, Maciej Chęciński, Kamila Chęcińska, Klaudia Kwiatkowska, Kalina Romańczyk, Adam Michcik, Barbara Wojciechowska, Tomasz Wach, Maciej Sikora

**Affiliations:** 1 Department of Oral Surgery, Preventive Medicine Center, 12 Komorowskiego Street, 30-106 Cracow, Poland; amelia.a.hoppe@gmail.com (A.H.); klaudia011998@wp.pl (K.K.); kalina.romanczyk@wp.pl (K.R.); 2 National Medical Institute of the Ministry of the Interior and Administration, 137 Wołoska Street, 02-507 Warsaw, Poland; maciej.checinski@pimmswia.gov.pl (M.C.); kamila.checinska@pimmswia.gov.pl (K.C.); sikora-maciej@wp.pl (M.S.); 3 Department of Maxillofacial Surgery, Hospital of the Ministry of the Interior and Administration, 51 Wojska Polskiego Street, 25-375 Kielce, Poland; 4 Department of Maxillofacial Surgery, Faculty of Medicine, Medical University of Gdansk, 17 Mariana Smoluchowskiego Street, 80-214 Gdansk, Poland; adam.michcik@gumed.edu.pl (A.M.); barbara.wojciechowska@gumed.edu.pl (B.W.); 5 Department of Maxillofacial Surgery, Medical University of Lodz, 251 Pomorska Street, 92-213 Lodz, Poland; tomasz.wach@umed.lodz.pl; 6 Department of Biochemistry and Medical Chemistry, Pomeranian Medical University, 72 Powstańców Wielkopolskich Street, 70-111 Szczecin, Poland

**Keywords:** temporomandibular joint, temporomandibular joint dislocation, temporomandibular joint disorders, mandibular dislocation

## Abstract

**Background/Objectives**: Children’s unique physiological and behavioral needs require individualized treatment planning. It seems reasonable to investigate treatment options for recurrent temporomandibular joint (TMJ) dislocation and assess their outcomes. This review was conducted with the purpose of identifying recent therapeutic approaches for TMJ dislocation in pediatric patients and evaluating their effectiveness. **Methods**: Searches were conducted on 21 September 2025, using BASE, PubMed, and Scopus. The review included studies with measurable outcomes, published between 2000 and 2025, that focused on patients under the age of 18 with recurrent TMJ dislocation. Studies with unclear diagnoses or undefined treatment methods were excluded. Risk of bias was evaluated using the Joanna Briggs Institute’s critical appraisal tool. The results were tabulated. **Results**: Based on the inclusion criteria, nine studies were included: one case-control study, three case series, and five case reports. Invasive treatment methods applied in pediatric patients were reported in two of those. Minimally invasive and conservative treatment methods were most frequently described, with botulinum toxin injections being the most commonly reported minimally invasive approach. **Conclusions**: Research revealed that conservative and minimally invasive methods are preferred in pediatric patients’ treatment. Due to the heterogeneity and limited number of available literature, consistent conclusions regarding the effectiveness of different treatment methods for recurrent TMJ dislocation in children could not be drawn. This study received no funding. PROSPERO ID number: CRD420251139493.

## 1. Introduction

### 1.1. Background

The temporomandibular joint (TMJ) is a synovial joint formed by the mandible, temporal bone, and articular disc. The articular head is situated on the condylar process of the mandible, while the temporal bone contains the socket of the joint formed by the glenoid fossa [1,2,3]. The articular disc divides the joint cavity into upper and lower compartments, both lined with fibrocartilage. A fibrocartilage coating plays an essential role in maintaining the durability of a joint [2,4,5]. Joint movements are enabled by muscles, supported by ligaments, and limited by the cranial anatomy [2,6].

During jaw opening, rotational movement is followed by inferior and anterior sliding of the head of the mandible, towards the articular eminence, eventually positioning itself over its top. This motion is facilitated by the activity of the suprahyoid muscles, along with the lateral pterygoid muscle [7,8]. Closure and retraction are executed by the masseter and temporalis. Protrusion is achieved by the synergistic action of medial and lateral pterygoid muscles [7,8,9].

Proper motor function of the temporomandibular joint relies on the precise coordination of all components within its highly complex structure; consequently, any even minor anatomical or neuromuscular disturbances can predispose to TMJ dysfunction. Dislocation of the TMJ, most often, is a result of wide mouth opening, such as during yawning or because of prolonged mouth opening during dental procedures, whereby the mandibular condyle moves beyond the glenoid fossa, becomes trapped, and cannot return to its original position without professional help [10,11,12,13]. The mandible can dislocate posteriorly, laterally, medially, or anteriorly, with anterior dislocation being the most common [12]. A distinction is made between acute, chronic, and recurrent luxation, and also between bilateral and unilateral luxation [12,13,14].

Various predisposing factors have been linked to spontaneous TMJ dislocation that may be associated with excessively flexible ligaments, anatomical irregularities, intense muscle activity, underlying neuromuscular dysfunction, connective tissue disorders, and some medications [11,13,14,15,16].

Emergency mandibular dislocation is usually addressed by manual repositioning of the condyle, using either a posterior or anterior approach [11,13,14,16]. However, this is a temporary solution. Patients with recurrent dislocations should be treated preventively [14,16].

Conservative treatment methods for TMJ dislocation include the use of jaw exercises, acrylic blocks, impression compound spacers, intermaxillary fixation (IMF), injection of botulinum toxin, sclerosing or prolotherapy, and occlusal splints [13,14,16,17,18]. Often, several forms of treatment are used simultaneously in therapy. For example, after applying a posterior acrylic bite block combined with the use of elastic traction, exercises will be recommended [17]. IMF, achieved by wiring the patient’s teeth, limits dislocation by ensuring immobilization. Thus, this method cannot be used in children without erupted teeth or in edentulous patients [19,20]. Botulinum toxin administered to the lateral pterygoid muscle inhibits the release of acetylcholine from the presynaptic membrane, weakening muscle contraction [6,21]. Prolotherapy is a technique involving the injection of irritants into a joint to provoke cell proliferation, with the aim of enhancing the mechanical stability of the surrounding ligaments [6,21].

Surgical methods involve approaches such as increasing the height of the eminence (e.g., Dautrey’s procedure), soft tissue surgery to restrict condylar movement, and eminectomy [16,22,23].

### 1.2. Rationale

In young children, the condylar process of the mandible has a rounded shape, and the glenoid fossa is flatter [24,25,26]. As they grow, the condylar process becomes longer and tilts forward, while the glenoid fossa increases in depth [25,26]. Such changes progress gradually throughout development as a result of mechanical forces generated by jaw movements [27,28].

Treating children can be challenging for doctors, as it requires adaptation to their stage of development. Effective treatment can also be impeded by a lack of cooperation from young patients. Children require a specific approach and a lot of patience. Still, performing certain procedures while keeping the child conscious may prove impossible.

### 1.3. Objectives

The primary objective of the study was to identify the main treatment approaches applied in patients under 18 years of age with recurrent temporomandibular joint dislocation, by comparing conservative, minimally invasive and invasive treatment methods and to determine the potential advantages associated with each approach.

## 2. Materials and Methods

This systematic review has been developed in accordance with the Preferred Reporting Items for Systematic Reviews and Meta-Analyses (PRISMA) methodology and registered in PROSPERO under the number CRD420251139493 [29]. The checklists for the report and abstract can be found in Supplementary Documents S1 and S2, respectively.

### 2.1. Eligibility Criteria

The eligibility criteria were established based on the PICOS methodology (Table 1). This review includes publications on patients under 18 years old and those including both children and adults with recurrent TMJ dislocation. However, studies in which TMJ dislocation was treated as part of broader therapy, as well as cadaver and animal studies, were excluded. All treatment methods outlined in research were included if performed during treatment and properly documented. Studies in which the level of evidence could not be determined, along with preprints and conference proceedings, were excluded. Then articles published within the current quarter-century (2000–2025) were selected for full-text screening. For the article to be included in the study, it must contain at least one of the following measures: dislocation frequency (pre-/post-treatment), acoustic symptoms (pre-/post-treatment), mandibular opening path (pre-/post-treatment), pain intensity (pre-/post-treatment) or range of abduction (pre-/post-treatment).

### 2.2. Search Strategy

The initial literature searches were undertaken using Bielefeld Academic Search Engine (BASE), PubMed and Scopus. The search was conducted on 21 September 2025. Search queries were formulated based on the eligibility criteria (Table 1), and after adjusting and refining, the final queries presented in Table A1 were used.

### 2.3. Selection and Data Processing

According to the PICOS criteria, research selection was performed using the Rayyan platform [22]. Duplicated articles were excluded, and the screening of abstracts began. 

In the first stage, two authors (K.C. and A.H.) independently assessed the titles and abstracts, selecting articles that met the eligibility criteria for inclusion in the full text assessment. All articles for which discrepancy occurred were included for full-text review. The same authors conducted a full-text assessment. Discrepancies identified at this stage were first addressed through discussion between the two assessing researchers; if consensus could not be achieved, the final decision was made by the third author.

Using a standard form developed in Google Sheets, part of the Google Workspace suite (2025 version, Google LLC, Mountain View, CA, USA), the following data were extracted from the included studies: (1) first author and publication year, (2) type of intervention, (3) patient characteristic (age, sex), (4) dislocation frequency before treatment, (5) dislocation frequency after treatment, (6) acoustic symptoms before treatment, (7) acoustic symptoms after treatment, (8) mandibular opening path before treatment, (9) mandibular opening path after treatment, (10) pain intensity before treatment, (11) pain intensity after treatment, (12) range of abduction before treatment, and (13) range of abduction after treatment. The collected data were organized into tables.

### 2.4. Study Risk of Bias Assessment

The risk of bias was evaluated using the Joanna Briggs Institute’s critical appraisal tool, which is designed to assess the quality of various research studies, with separate checklists tailored to different study designs [30]. Thus, the JBI checklists for case–control studies, case reports and case series were used, depending on the study type. This assessment was conducted independently by two reviewers, A.H. and M.C. The (1) percentage agreement, (2) Cohen’s kappa, (3) PABAK, (4) AC1 Gweta were calculated to measure the inter-reviewer agreement. Any discrepancies between the reviewers were resolved through discussion until consensus was reached.

### 2.5. Qualification of Reports

Based on the Oxford Centre of Evidence-Based Medicine 2011 Levels of Evidence scale, the authors assessed the level of evidence for each study. Due to the very limited number of high-quality articles, studies were included regardless of their level of evidence, as long as evaluation was possible.

## 3. Results

**Figure 1:**
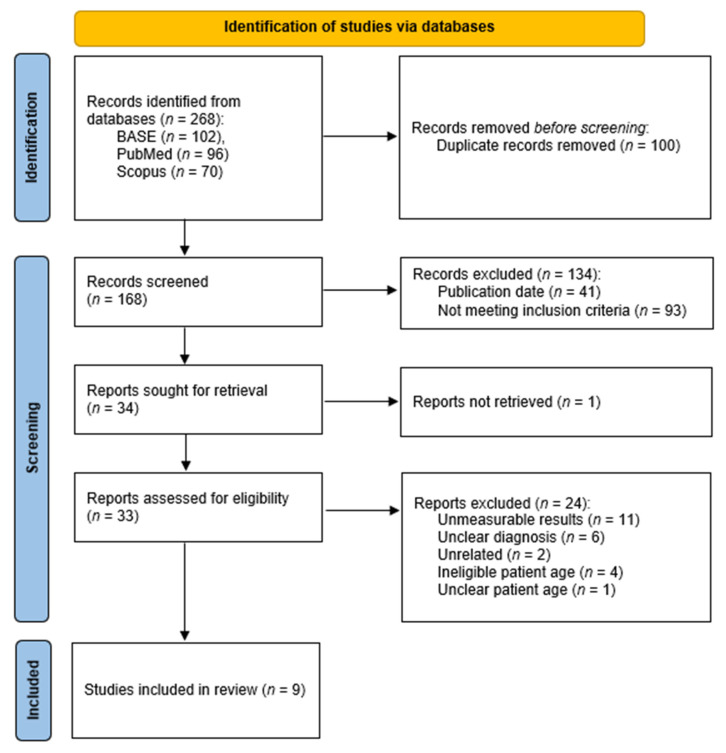
PRISMA flow diagram.

In total, 268 articles were identified through the search: 102 via BASE, 96 from PubMed, and 70 from Scopus. After removing duplicates, 168 records remained for screening, out of which 34 qualified for full-text evaluation. Unfortunately, despite efforts, access to the full text of one article could not be obtained [31]. Ultimately, after excluding 24 reports (Table A2), nine studies were included for the synthesis (Figure 1).

This study includes 5 case reports, 3 case series, and 1 case–control study. An assessments of level of evidence, along with the methods performed in each study, is presented in Table 2.

Table 3 presents the dislocation frequency before and after treatment as reported in all included studies, along with demographic data such as age and sex. The remaining extracted data, which could be classified under one of the considered measures, are presented in Table 4. Data from two patients across two different studies were excluded: in one case [33], the patient’s data were not classified into one of the measures, and in the other [38], the patient belonged to the control group. Since the mandibular opening path, initially planned as an assessment measure, was not examined in any of the analyzed studies, it was removed from the final data organization.

### Risk of Bias in Studies

The detailed risk of bias assessment for each study is summarized in Table A3, Table A4 and Table A5. The study by Triantafillidou et al. demonstrates a low risk of bias, as all criteria from the critical appraisal tool were met [38]. The case series by Coser et al., Yoshida et al., and Gadre et al. present a moderate overall risk of bias [34,36,39]. Although each of these studies fulfilled the majority of the criteria, they consistently lacked appropriate statistical analysis. Additionally, issues related to unclear reporting of participant inclusion and, in some cases, measurement reliability, may further affect the validity and generalizability of their findings. The case reports were assessed as having varying levels of risk of bias [32,33,35,37,40]. Only the report by Stark et al. met all the appraisal criteria, indicating a low risk of bias [37]. The remaining case reports were assessed as having a moderate risk of bias due to incomplete reporting in key areas [32,33,35,40]. The agreement between the two authors independently assessing the risk of bias was high, with approximately 78% overlap in responses. Despite this, Cohen’s kappa coefficient was relatively low (≈0.10). This discrepancy can be explained by the fact that both reviewers frequently selected the “Yes” option, which artificially inflated the expected chance agreement. Therefore, the low kappa value in this case does not indicate substantial discrepancies in the assessment of bias risk, but rather reflects an uneven distribution of responses across the options (“Yes”, “No”, “Unclear”). For comparison, alternative indicators of agreement were calculated, demonstrating moderate to high concordance between the reviewers (PABAK = 0.56; AC1 Gweta = 0.71).

## 4. Discussion

TMJ dislocations are a part of TMJ disorders (TMDs), a collective term that also includes conditions such as arthralgia, degenerative joint disease, osteoarthritis, and other pathologies associated with TMJ dysfunctions [41]. The latest research showed that TMDs may affect even 31% of adults and 11% of children [42]. Furthermore, Zielinski et al. estimate that by 2050, this prevalence could increase to 44% of the global population and 37% of the European population [43]. Despite this, the available data concerning the incidence of TMJ dislocations in children, particularly recurrent dislocations, is notably limited and primarily derived from case reports. Mandibular dislocations are reported slightly more frequently in women and tend to occur most commonly in the fourth and fifth decades of life. According to Papoutsis et al., the majority of TMJ dislocations are recurrent (62.5%) [44]. Similar results were reported by Tarhio et al. (61.9%) [14].

Current research shows that there is no single factor responsible for TMDs, as their etiology is multifactorial [41]. In addition to the anatomical variations in bone structure observed in pediatric patients, such as a flatter glenoid fossa, various other conditions may predispose to TMJ dislocation, including connective tissue disorders like Ehlers-Danlos Syndrome, which are associated with structural defects in collagen and elastin leads to loss of stability and ligament support [45,46]. Additionally, neuromuscular diseases, such as muscular dystrophies, due to myotonia and weakness of masticatory muscles, result in impaired mandibular movements, thereby increasing the load on the temporomandibular joint, as well as neurological disorders that result in seizures or involuntary movements, can further increase the risk [47,48]. Chronic steroid therapy is another contributing factor, as it may lead to muscle and ligament weakening, thereby promoting the occurrence of recurrent dislocations [49,50]. Another condition that may prove crucial in TMD development is joint hypermobility, a prevalent condition in childhood, affecting between 8% to 39% of school-aged individuals [51]. The benign form most commonly affects individuals between the ages of 3 and 10, with its prevalence diminishing as children age [52]. Notably, hypermobile joints are more vulnerable to mechanical stress and may experience excessive wear over time. Furthermore, patients with joint hypermobility are more likely to develop TMDs in later stages of life [53].

It is worth noting that in children, the majority of TMJ dislocations are associated with trauma [14]. Therefore, recurrent TMJ dislocations in pediatric patients are especially rare. Regardless of age, TMJ dislocations occurring repeatedly during daily activities should be managed starting with the identification and elimination of underlying causes, where feasible. Then, the most appropriate therapeutic strategy should be selected from available methods.

The results of our research highlighted that among pediatric patients with recurrent TMJ dislocation, conservative and minimally invasive treatment methods are most commonly applied. However, the results of such treatment were not documented reliably enough to confirm or deny their effectiveness. Considering the low risk associated with this approach, such methods should be considered as first-line treatment options. Particularly considering the nature of treating pediatric patients, it seems reasonable to avoid needle-based interventions, thereby supporting the preference for conservative treatment methods. Notably, several reports indicated that successful recovery often requires the involvement of various specialists, including psychologists, neurologists, and others. This multidisciplinary approach results from the multifactorial nature of TMJ dislocation and the incomplete understanding of the pathophysiological mechanisms that contribute to its development.

It is worth noting that lack of cooperation and significant distress in pediatric patients can be managed with the administration of nitrous oxide or various forms of sedation (e.g., propofol-induced). Patient discomfort can be alleviated with local anesthesia. Additionally, these medications may be beneficial in cases of unsuccessful jaw repositioning maneuvers, as increased tension of the masticatory muscles impedes completion of the procedure. The use of nitrous oxide prior to the repositioning maneuver was reported by Mohan et al. and Sicard et al., and prior to the botulinum toxin injections by Stark et al. [32,33,37]. Light intravenous sedation before manual reduction was described by French et al., while propofol sedation was reported by Sicard et al. [33,35]. Coser et al. and Stark et al. described injections of local anaesthetic administered before arthrocentesis and prior to carrying out the repositioning maneuver, respectively [36,37]. As the research revealed, sedation alone may in some cases be sufficient, Sicard et al. reported spontaneous reduction, which occurred under propofol-induced sedation [33].

Botulinum toxin injections were the most commonly reported minimally invasive method, with autologous blood injections reported nearly as frequent. Botulinum toxin type A, which is produced by the gram-positive bacterium *Clostridium botulinum*, exerts a paralytic effect by rapidly binding to presynaptic cholinergic nerve terminals [34]. Typically, injecting into the lateral pterygoid muscle reduces excessive muscular activity, contributing to recurrent dislocation [54]. Autologous blood injections stimulate a localized inflammatory response, contributing to enhanced joint stability and the promotion of fibrosis. Clinical follow-up assessments of patients who have undergone this treatment demonstrate no radiographic or functional evidence of ankylosis [6,55]. Conservative treatment is commonly adopted for children with TMJ dislocation due to its less invasive nature and better psychological tolerance, while ensuring ongoing craniofacial growth is not compromised.

It should also be emphasized that the available literature lacks sufficient long-term evidence to support the choice of surgical interventions in children. There are several important considerations regarding children that make this approach more complex and potentially controversial, which include ongoing growth, joint physiology, and potential complications like joint ankylosis [56]. Immobilization can impair normal growth, reduce the flow of synovial fluid, and impede nutrient exchange. Therefore, early mobilization and physiotherapy are advocated for successful treatment [57,58]. Invasive treatment methods were reported only in two of the included studies [35,39]. Nevertheless, in both cases, surgical procedures were chosen as a last resort and resulted in successful outcomes. Thus, high-quality, reliable further research using valid and comparable measures should be prioritized in the future. 

Considering all of the limitations, due to the lack of comparable reviews available in recent years, it is not feasible to draw comparisons with existing literature. The level of evidence of included studies is generally low, with only four publications rated above level 5, introducing a high risk of bias. Over the past quarter-century, only nine articles on this topic have been found, highlighting an unsatisfactory level of scientific attention. As the review revealed a lack of sufficient literature, it seems reasonable to recommend conducting thorough studies of any kind on this topic. However, in order to maintain high-quality data organization and comparability, standardized outcome measures for treatment effectiveness should be developed. Thus, authors intending to conduct future research should consider assessing and reporting at least all of the above: dislocation frequency, occurrence of acoustic symptoms, mandibular opening path, pain intensity, and range of jaw abduction, both before and after the investigated treatment method.

Consequently, although conservative and minimally invasive methods are more frequently chosen for the treatment of pediatric patients, the current literature does not indicate that age alone should be considered a sufficient contraindication to surgical approaches in children, due to a lack of evidence. The significance of an interdisciplinary approach seems to be highlighted, as this treatment strategy appears repeatedly in successfully resolved case reports. However, the available data are limited and lack the consistency needed to draw any definitive conclusions.

The main limitations identified while conducting the study were primarily the small number of eligible studies available in the literature and the particularly small number of participants involved in the included research. In addition, the overall level of evidence was low, as most of the included articles were case reports. Consequently, heterogeneity was observed among study designs, interventions, and outcome measures, which made direct comparisons difficult or impossible. Furthermore, the possibility of reporting bias among the included studies cannot be fully excluded. The limitations mentioned above suggest that the results should be interpreted with caution.

In conclusion, although research indicates that conservative and minimally invasive methods are the most frequently chosen approaches for managing recurrent temporomandibular joint dislocation in children, the limited number of recent studies on this topic prevents drawing definitive conclusions regarding their effectiveness. While some reports suggest that surgical approaches may also be effective, they should be considered a last resort, and further high-quality research is needed to confirm their safety in the paediatric population. Importantly, future studies should employ the outcome measures identified in this review to ensure comparability and consistency of results.

## Figures and Tables

**Table 1:** Eligibility criteria.

Domain	Inclusion Criteria	Exclusion Criteria
Patient	Patients younger than 18 years old presenting recurrent TMJ dislocation	Cadaver and animal studies
Intervention	Any treatment method	Unclear diagnosis, unknown treatment method, TMJ dislocation treatment as part of a broader therapy
Comparison	Any or none	Not applicable
Outcomes	At least one of the required symptoms measures	None of required
Settings	Research published in scientific journals	Preprints, conference proceedings, papers published before 2000

**Table 2:** Included studies.

First Author, Publication Year	Conservative Treatment Methods	Minimally Invasive Treatment Methods (Injections)	Invasive Treatment Methods	Level of Evidence
Mohan, 2022 [32]	Hippocratic jaw reduction, IMF, splint, pharmacotherapy, head gear	botulinum toxin injection with Stimuplex^®^ (B. Braun Melsungen AG, Melsungen, Germany) nerve stimulator		level 5
Sicard, 2018 [33]	Nelaton manoeuver, chin to vertex immobilization, another similar immobilization device, pharmacotherapy, psychological assessment			level 5
Yoshida, 2018 [34]		autologous blood injection		level 4
French, 2017 [35]	manual repositioning, IMF, pharmacotherapy for facial tic disorder	botulinum toxin injection	eminectomy	level 5
Coser, 2015 [36]		arthrocentesis, autologous blood injection		level 4
Stark, 2015 [37]	manual repositioning, pharmacotherapy	botulinum toxin injection		level 5
Triantafillidou, 2012 [38]		autologous blood injection		level 3
Gadre, 2010 [39]			Dautrey’s procedure	level 4
Martínez-Pérez, 2004 [40]		botulinum toxin injection		level 5

IMF—intermaxillary fixation.

**Table 3:** Research results across groups of pediatric patients and results regarding dislocation frequency before and after treatment.

First Author, Publication Year	Age, Sex	Dislocation Frequency Before Treatment	Dislocation Frequency After Treatment
Mohan, 2022 [32]	10 years, male	During the 1-month observation period, the patient experienced 5 episodes.	Conservative treatment methods proved unsuccessful. Then, minimally invasive treatment methods were applied, but a luxation episode occurred 6 months later. Subsequently, the patient was provided with interdisciplinary care and pharmacotherapy, after which no further episodes were observed.
Sicard, 2018 [33]	26 months, male	More than 20 dislocation episodes occurred within 2 months.	Conservative methods provided with psychological care resulted in 2 dislocation episodes over the following 6 months.
Yoshida, 2018 [34]	17 years, female	The frequency prior to the intervention was not reported.	No episodes were reported during the 36 months after treatment.
French, 2017 [35]	14 years, male	The frequency prior to the first intervention was not reported.	After conservative treatment, dislocation occurred multiple times. Then, minimally invasive treatment methods were introduced, resulting in a one-month asymptomatic period. An alternative conservative method was applied; however, it was ineffective in the long term. Minimally invasive treatment was performed every three months, resulting in no luxation for 18 months. For the duration of one year, the patient was unsuccessfully treated using minimally invasive methods combined with interdisciplinary care. Ultimately, an eminectomy was performed, and no luxation episodes were reported at the six-month follow-up appointment.
Coser, 2015 [36]	15 years, female	Over the course of one year, 4 episodes occurred, with at least 3 taking place within a 6-month period, meeting the inclusion criteria.	No episodes were reported during the 33 months after treatment.
Stark, 2015 [37]	9 years, female	The patient experienced more than 40 dislocations over a two-week period.	Initially applied conservative methods proved ineffective. Despite the subsequent use of minimally invasive methods, the patient experienced recurring luxations for 4 months. The treatment was repeated, but after another 4 months, luxations recurred. However, over the next 9 months, no episodes were reported.
Triantafillidou, 2012 [38]	16 years, female	The frequency prior to the intervention was not reported.	The frequency following the intervention was not reported.
Gadre, 2010 [39]	17 years, female	The frequency prior to the intervention was not reported.	No episodes were reported during the 36 months after treatment.
	14 years, male	The frequency prior to the intervention was not reported.	No episodes were reported during the 28 months after treatment.
Martínez-Pérez, 2004 [40]	17 years, female	The frequency prior to the intervention was not clearly reported.	A total of 4 injections were administered over the course of one year, during which the patient experienced recurring luxations. Following the final injection, the patient became asymptomatic.

**Table 4:** Research results in groups of pediatric patients, remaining outcome measures.

First Author, Publication Year	Acoustic Symptoms Before Treatment	Acoustic Symptoms After Treatment	Pain Intensity Before Treatment	Pain Intensity After Treatment	Range of Abduction Before Treatment [mm]	Range of Abduction After Treatment [mm]
Coser, 2015 [36]	N/S	N/S	N/S	N/S	51	48
Stark, 2015 [37]	N/S	N/S	Significant	None	Restricted because of pain	N/S
Triantafillidou, 2012 [38]	Severe	Mild	N/S	N/S	50	45
Gadre, 2010 [39]	N/S	N/S	N/S	N/S	47	51
	N/S	N/S	N/S	N/S	42	28

N/S—not specified.

## Data Availability

Data is contained within the article or Supplementary Materials.

## Supplementary Materials

The following supporting information can be downloaded at: https://www.mdpi.com/article/10.3390/jcm14217881/s1, Document S1: PRISMA 2020 Checklist; Document S2: PRISMA 2020 for Abstracts Checklist.

## Abbreviations

The following abbreviations are used in this manuscript:

TMJ	Temporomandibular joint
TMDs	Temporomandibular joint disorders
IMF	Intermaxillary fixation

## Appendix A

**Table A1:** Search queries.

Database	Search Query
BASE	(habitual OR recurrent OR chronic OR repeated OR relapsing OR recurring) AND (dislocation OR luxation OR subluxation OR displacement OR malposition) AND (temporomandibular OR mandible OR TMJ OR jaw) AND (child OR kids OR minor OR adolescent OR toddler OR preteen OR juvenile OR pediatric OR underage OR preadolescent OR youth) AND (treatment OR management OR therapy OR remediation OR remedy OR care OR approach)
PubMed	(habitual OR recurrent OR chronic OR repeated OR relapsing OR recurring) AND (dislocation [Title] OR luxation [Title] OR subluxation [Title] OR displacement [Title] OR malposition [Title]) AND (temporomandibular OR mandible OR TMJ OR jaw) AND (child OR kids OR minor OR adolescent OR toddler OR preteen OR juvenile OR pediatric OR underage OR preadolescent OR youth) AND (treatment OR management OR therapy OR remediation OR remedy OR care OR approach)
Scopus	TITLE-ABS-KEY ((habitual OR recurrent OR chronic OR repeated OR relapsing OR recurring) AND (temporomandibular OR mandible OR tmj OR jaw) AND (child OR kids OR minor OR adolescent OR toddler OR preteen OR juvenile OR pediatric OR underage OR preadolescent OR youth) AND (treatment OR management OR therapy OR remediation OR remedy OR care OR approach)) AND (TITLE (dislocation OR luxation OR subluxation OR displacement OR malposition))

**Table A2:** Studies excluded at full-text assessment stage.

First Author, Publication Year	Digital Object Identifier (DOI) or Alternative	Reason for Exclusion
Souza, 2025	10.1080/08869634.2022.2110190	Impossible to determine which patients were under the age of 18.
Lee, 2024	10.21203/rs.3.rs-5223475/v1	No treatment methods described.
Wang, 2021	10.1097/MD.0000000000024012	Dislocation was a one-time occurrence; there was no recurrence.
Aamir, 2020	-	Impossible to determine which method was performed on the pediatric patient.
Bukhari, 2020	10.5455/JPMA.5002	Impossible to determine if the study contained patients under 18 and which method was performed.
Papoutsis, 2018	10.2147/OAEM.S174116	Impossible to determine which method was performed on the pediatric patient.
Gorchynski, 2014	10.1016/j.jemermed.2014.06.050	Patients aged 18 years or older.
Oshiro, 2014	10.1016/j.jcms.2014.04.018	None of the required measures.
Ungor, 2013	10.1097/SCS.0b013e31827ff14f	Impossible to determine which results describe pediatric patients.
Ybema, 2013	10.1016/j.ijom.2012.09.017	Impossible to recognize any of the required measures in a pediatric patient.
Zhou, 2013	10.1016/j.bjoms.2013.08.018	Impossible to determine which results describe pediatric patients.
Bekreev, 2012	PMID: 23268183	Unrelated topic.
Torres, 2012	10.1016/j.ijom.2012.03.008	Impossible to determine which method was performed on the pediatric patient.
Zakariaei, 2012	PMID: 22418992	Dislocation was a one-time occurrence; there was no recurrence.
Chhabra, 2011	10.4103/0970-4388.90758	Dislocation was a one-time occurrence; there was no recurrence.
Kummoona, 2010	10.1097/SCS.0b013e3181f3c682	Patients aged 18 years or older.
Papadopoulos, 2010	10.1016/j.ijom.2009.12.011	Dislocation was a one-time occurrence, there was no recurrence.
Sang, 2010	10.4314/eamj.v87i1.59949	Unclear if the patient has recurrent TMJ dislocation; impossible to determine which method was performed on the pediatric patient.
Machon, 2009	10.1016/j.joms.2008.08.044	Impossible to recognize any of the required measures in a pediatric patient.
Medra, 2008	10.1016/j.bjoms.2007.08.004	Patients aged 18 years or older.
Willemsen, 2008	PMID: 18188830	Unrelated topic.
Cardoso, 2005	10.1016/s1808-8694(15)31282-9	Patients aged 18 years or older.
Ugboko, 2005	10.1016/j.ijom.2004.10.025	Impossible to determine if the pediatric patient has recurrent TMJ dislocation; impossible to determine which method was performed on the pediatric patient.
Nitzan, 2002	10.1053/joms.2002.31846	Impossible to recognize any of the required measures in a pediatric patient.

**Table A3:** Critical appraisals/case–control studies.

First Author, Publication Year	Were the Groups Comparable Other than the Presence of Disease in Cases or the Absence of Disease in Controls?	Were Cases and Controls Matched Appropriately?	Were the Same Criteria Used for Identification of Cases and Controls?	Was Exposure Measured in a Standard, Valid and Reliable Way?	Was Exposure Measured in the Same Way for Cases and Controls?	Were Confounding Factors Identified?	Were Strategies to Deal with Confounding Factors Stated?	**Were Outcomes Assessed in a Standard, Valid and Reliable Way for Cases and Controls?**	**Was the Exposure Period of Interest Long Enough to Be Meaningful?**	**Was Appropriate Statistical Analysis Used?**
Triantafillidou, 2012 [38]	Yes	Yes	Yes	Yes	Yes	Yes	Yes	Yes	Yes	Yes

**Table A4:** Critical appraisal, case series.

First Author, Publication Year	Were There Clear Criteria for Inclusion in the Case Series?	Was the Condition Measured in a Standard, Reliable Way for All Participants Included in the Case Series?	Were Valid Methods Used for Identification of the Condition for All Participants Included in the Case Series?	Did the Case Series Have Consecutive Inclusion of Participants?	Did the Case Series Have Complete Inclusion of Participants?	Was There Clear Reporting of the Demographics of the Participants in the Study?	Was There Clear Reporting of Clinical Information of the Participants?	Were the Outcomes or Follow-Up Results of Cases Clearly Reported?	Was There Clear Reporting of the Presenting Site(s)/Clinic(s) Demographic Information?	Was Statistical Analysis Appropriate?
Yoshida, 2018 [34]	Yes	Unclear	Yes	Yes	Unclear	Yes	Yes	Yes	Yes	No
Coser, 2015 [36]	Yes	Yes	Yes	Yes	Unclear	Yes	Yes	Yes	Yes	No
Gadre, 2010 [39]	Yes	Yes	Unclear	Yes	Unclear	Yes	Yes	Yes	Yes	No

**Table A5:** Critical appraisal, case reports.

First Author, Publication Year	Were the Patient’s Demographic Characteristics Clearly Described?	Was the Patient’s History Clearly Described and Presented as a Timeline?	Was the Current Clinical Condition of the Patient on Presentation Clearly Described?	Were Diagnostic Tests or Assessment Methods and the Results Clearly Described?	Was the Intervention(s) or Treatment Procedure(s) Clearly Described?	Was the Post-Intervention Clinical Condition Clearly Described?	Were Adverse Events (Harms) or Unanticipated Events Identified and Described?	Does the Case Report Provide Takeaway Lessons?
Mohan, 2022 [32]	Yes	Yes	Yes	Yes	Yes	Yes	No	Yes
Sicard, 2018 [33]	Yes	Yes	Yes	Yes	Yes	Yes	No	Yes
French, 2017 [35]	Yes	Unclear	Yes	Yes	Yes	Yes	Yes	Yes
Stark, 2015 [37]	Yes	Yes	Yes	Yes	Yes	Yes	Yes	Yes
Martínez-Pérez, 2004 [40]	Yes	Unclear	Yes	Unclear	Yes	Yes	No	Yes
